# Mario Ageno and the status of biophysics

**DOI:** 10.1007/s40656-024-00617-7

**Published:** 2024-05-24

**Authors:** Daniele Cozzoli

**Affiliations:** Pompeu Fabra University, Barcelona, Spain

**Keywords:** Biophysics, Molecular biology, Quantum mechanics, Bacteriophage, Virus

## Abstract

This essay focuses on Mario Ageno (1915–1992), initially director of the physics laboratory of the Italian National Institute of Health and later professor of biophysics at Sapienza University of Rome. A physicist by training, Ageno became interested in explaining the special characteristics of living organisms origin of life by means of quantum mechanics after reading a book by Schrödinger, who argued that quantum mechanics was consistent with life but that new physical principles must be found. Ageno turned Schrödinger’s view into a long-term research project. He aimed to translate Schrödinger’s ideas into an experimental programme by building a physical model for at least a very simple living organism. The model should explain the transition from the non-living to the living. His research, however, did not lead to the expected results, and in the 1980s and the 1990s he focused on its epistemological aspect, thinking over the tension between the lawlike structure of physics and the historical nature of biology. His reflections led him to focus on the nature of the theory of evolution and its broader scientific meaning.

## Quantum mechanics and the special characteristics of living organisms

Mario Ageno was an interesting figure in the panorama of post-war scientific research in Italy because of his original epistemological perspective on the conceptual relations between biological and physical sciences. He was, indeed, one of the few researchers who engaged with the question of the special characteristics of living organisms. Soon after graduating in physics in 1936 with a final dissertation on slow neutrons with Enrico Fermi as his supervisor, Ageno started working in the laboratory of physics of the ISS (Istituto Superiore di Sanità), the national institute of health (Amaldi, 1989). In 1947, he became interested in the relations between quantum mechanics and the origin of life, when he translated into Italian Erwin Schrödinger’s book *What is Life?* Throughout his career Ageno aimed to show that quantum mechanics was consistent with life, developing Schrödinger’s perspective in an experimental programme. Later, he also became interested in Darwinism, integrating his perspective with the new synthetic evolutionary theory. While one may consider Ageno’s contribution as far from world– breaking, his effort to bridge the gap between physical and life sciences paid off later in his life, when he was acknowledged internationally for stimulating a debate on these issues, also developing a virtually unique career trajectory.

Ageno’s contribution to a subject that had its roots in exchanges between Bohr and Schrödinger, among others, remains relatively obscure. This obscurity can be attributed to the fact that a substantial portion of his work was published in Italian and his research lacked the transformative impact on the field of molecular biology that figures like Max Delbrück, for example, achieved. Furthermore, Ageno’s perspective on biophysics failed to garner the attention of Italian policymakers, because it conflicted with that of molecular biologists.

In the subsequent section we will reconstruct the debate on quantum mechanics and life in the writings of Bohr and Delbrück, highlighting its importance for the inception of molecular biology. Section 3 will be dedicated to Schrödinger’s stance and how Ageno further developed it. Moving forward, Sect. 4 will scrutinize the 1960s debate between Ageno and Wigner on quantum mechanics and the origin of life. Section 5 will centre on Ageno’s reflection on biophysics as the physics of living systems, a perspective that set him at odds with the community of molecular biologists and fostered a distinct interest in Darwinism. The final section will offer a comprehensive evaluation of Ageno’s contribution to biophysics and the debate on quantum mechanics and life.

## Ageno’s encounter with Schrödinger

In 1927, Niels Bohr formulated the principle of complementarity, according to which, properties of waves and particles had to be considered as complementary aspects of reality. Bohr’s view on the relations between physics and biology was grounded in his principle of complementarity. At a conference in 1932, he claimed that life should be accepted as an elementary fact, a starting point in biology like a quantum of action in physics, which from the point of view of classical mechanics is irrational. In physics, classical mechanics cannot explain the stability of atoms, therefore one must accept quantum postulates. Likewise, in biology current chemistry and physics seem unable to explain life, therefore one must accept it as a primitive fact (Bohr, 1933; Kay, 1992, 1993, p. 133; Leyla, Freire & El Hani, 2015; Morange, 2020, p. 72; see also McKaughan, 2005). Bohr’s view remained on a philosophical level: he did not translate it into a research programme (Leyla et al., 2015). It is worth noting that once life has been postulated as a primitive fact, it is hard to imagine how it could be explained by means of more fundamental physical principles.

Bohr’s ideas intrigued Max Delbrück, a young physicist who had spent six months in Bohr’s laboratory (Delbrück, 1986, p. 1; Kay, 1993, p. 133; McKaughan, 2005). In 1937 Delbrück migrated to the USA and also “migrated” from physics to biology (Leyla et al., 2015, p. 243). Delbrück attended Bohr’s conference on *Light and Life*, and often acknowledged that Bohr played a part in his choice to devote himself to biology (Delbrück, 1949, 1963 and, 1976; see also Kay, 1992; McKaughan, 2005; Leyla et al., 2015, p. 243). Several years later, Delbrück remembered that the ultimate effect of Bohr’s lecture was to change the course of his life (Delbrück, 1976). Delbrück took the principle of complementarity as a source of analogies in biology (Kay, 1992; Leyla et al., 2015, p. 243). He, Nikolaï Timoffeev-Ressovsky and Karl G. Zimmer applied to the study of genes the approach of nuclear physics of bombarding the nucleus: they irradiated *Drosophilae* with X-rays and studied the genetic mutations that were induced. They formulated a quantum model for the gene: mutation was seen as the passage from one stable state to another (Morange, 2020, p. 42; Sloan & Fogel, 2011). However, Delbrück felt that he had not really progressed in his project of translating Bohr’s perspective into an experimental programme to solve the problem of life. In the following years, he left research on phage to his students and focused on another model, the *Phycomyces*, hoping to find a biological version of Bohr’s complementarity principle (Kay, 1992, pp. 12–13).

The research of Delbrück and Timoffeev-Ressovsky also attracted the attention of Erwin Schrödinger. Schrödinger hoped that eventually the causality of classical mechanics would be restated by the formulation of new principles. His view on life differed from that of Bohr, as it entailed that scientists should look for new physical principles (Fox Keller, 1990). The matter is, however, more intricate, as Bohr’s and Schrödinger’s views were sometimes mixed up. As Daniel J. McKaughan has convincingly argued, Stent, Watson and other pioneers of molecular biology tended to interpret Bohr’s *Light and Life* and Delbrück’s insistence on the principle of complementary as the expectation of finding new physical principles. Stent remembered that he found it difficult to understand Delbrück’s view on the complementarity principle. It is worth noting that Schrödinger’s approach entailed that studying biology would also lead to advancement in physics (McKaughan, 2005; see also Kay, 1993).

In 1947, Ageno translated Schrödinger’s popular book *What is Life?* into Italian. As he himself often recalled in the course of his life, the encounter with Schrödinger was a revelation. What Ageno found in Schrödinger was a life-long research programme. Instead of giving answers, Schrödinger formulated fundamental questions for physicists. He compared the discrete units of evolution, genes, to discrete quantum leaps. A quantum leap– claimed Schrödinger– can explain the presence of an allele in a gene locus. Schrödinger also observed that Delbrück’s model was consistent with the laws of quantum mechanics but lacked a physical explanation, and that a full understanding of it required further investigation in physics and new physical principles.^1^ He thought that quantic uncertainty played no role at all in spatiotemporal events inside a biological organism (Schrödinger, 1944). Delbrück reviewed Schrödinger’s book. He stressed the opposition between Schrödinger and Bohr, but concluded cautiously that:

Physicists and biologists who are not familiar with Bohr’s subtle complementarity argument will be inclined to take the physical nature of cellular processes for granted at the outset, and may be dissatisfied because Schrödinger does not advance our understanding of cellular mechanisms in any specific respect. The reviewer believes, however, that Schrödinger’s discussion of the types of laws of nature might exert a clarifying influence in biological thinking (Delbrück, 1945).

According to Evelyn Fox Keller, Schrödinger’s book had only indirect influence on scientists:

In retrospect, one would have to say not only that Schrödinger did not solve the problem of life, but that the paradox he identified as lying at the heart of this problem effectively dissolved in the face of subsequent developments. Nor can his book be said to have provided any actual suggestions for further research that proved to be useful (Fox Keller, 1990, p. 403).

Ageno was one of the few researchers who tried to elaborate an experimental programme from Schrödinger’s theses. Ageno endorsed Schrödinger’s view in a paper published some years later, in which he commented on the apparent non-applicability of the Second Law of thermodynamics to the living world. This was the question that Schrödinger had posed in his book.^2^ If the principle were not valid, a living organism would be able to get warmer by absorbing heat from a colder environment, which is contrary to everyday experience. Nonetheless, the principle states that a physical system evolves naturally towards a less ordered state, whereas more complex living organisms seem to be more ordered than simpler organisms. Ageno recalled the discussions in the Vienna Circle on the precision of scientific language with regard to common language. The notion of ‘order’ and ‘probability’ were quite different in common language and in statistics. The statistical probability of a state is a function of the state of the system. The order of a system is strictly connected to the probability of the state. The more ordered a state is, the fewer are the ways in which it could occur, so the relative thermodynamic probability is lower. Following Schrödinger, Ageno argued that the Second Law of thermodynamics is consistent with life. Considered as a physical system, a living organism receives energy in the form of solar radiation and transforms it by increasing its entropy level before giving it back to the exterior, so the living organism decreases its own entropy. To the physicist, the Second Law of thermodynamics seems to be inconsistent with life, because it is difficult to understand the limit of an organism as a physical system: one must study all the possible interactions. However, Ageno observed that the atoms in living organisms are arranged in systems that are too complex to be studied, at least until now. Ageno wanted to meet the challenge that Schrödinger had posed to physicists. He concluded his 1951 paper by claiming that Schrödinger was right: our physical theories do not have to be revised but we have to add to them new physical principles to account for the living world (Ageno, 1951).

Until 1959 Ageno’s grand project remained on a rather philosophical level. In 1949 he won two public competitions, one for a chair in theoretical physics at the University of Cagliari and one for the position of assistant in the physics laboratory of the ISS, at that time directed by Giulio Cesare Trabacchi. Quite surprisingly he chose the less prestigious position of assistant at the ISS to that of full professor, claiming that his bad health made him reluctant to leave Rome. He had, in fact, been seriously wounded during the war and remained in hospitals and rehabilitation centres until 1948.^3^ Several years later, Ageno also justified his choice by claiming that the focus of his research had shifted from physics to biophysics (‘Elenco delle pubblicazioni’, Archives of the Department of Physics of Sapienza University of Rome, Ageno Archive (hereinafter AA), box 1, folder 1). Indeed, had Ageno gone to a faculty of physics, he would have been making it more difficult for himself to pursue his inter-disciplinary interests. At the ISS, on the other hand, he was at the centre of physical and biomedical research in Italy and had plenty of resources, in addition to being able to study what he really liked and develop his ideas on the relations between physics and biology. It is also worth noting that Domenico Marotta, the director of the ISS, was transforming an institute devoted to public health into a major scientific institution. Marotta hired a Nobel laureate, Ernst Boris Chain, and a future Nobel laureate, Daniel Bovet. Chain directed a centre of microbiological research associated with a penicillin pilot plant that became the model for the research centre he created at Imperial College in the mid-1960s (Capocci, 2011; Cozzoli & Capocci, 2011; Cozzoli, 2015).

## Ageno and the debate on quantum mechanics and the origin of life in the 1960s

On Trabacchi’s retirement in 1959, Ageno became head of the ISS’s laboratory of physics and reoriented his research from nuclear physics to the physical foundations of biology. The 1943 Delbrück–Luria experiment had proved that bacteria are subject to genetic mutations, giving birth to bacterial genetics (De Chadarevian, 2002; Morange, 2020, pp. 53–56). After the discovery of the double helicoidal structure of DNA, molecular biology became the study of the functional and structural features of genetic material (Corbellini, 1997). The study of life focused on one component of the cell: the genes, and molecular biologists looked at life as the information or instructions encoded in genes (Fox Keller, 1990).

Ageno was, however, disappointed by the development of molecular biology. In 1987, he recalled that the successes of molecular biology had prevented a full development of the integration between biology and physics and a full understanding of the phenomenon of life after the pioneering work of D’Arcy Thompson and Norbert Wiener (Ageno, 1987, pp. 35–37). Molecular biology relegated physics to an ancillary role, a set of techniques suitable for studying molecular interactions and transformations (Ageno, 1987, IX–X). For Ageno, molecular biologists studied an organism as mere “games of molecules”, without understanding the huge complexity of the transition from molecular transformations to the living organism. Ageno aimed to develop a physical model suitable for studying a simple type of organism and then use that model to understand the transition from the non-living to the living (Ageno, 1987, p. X) Ageno’s research focused initially on phages (see M. Ageno ‘Elenco delle pubblicazioni’, in AA, box 1, folder 1). Phages had attracted the interest of scientists after the work of Salvador Luria and Max Delbrück, who had made them the core of their research and one of the main subjects of the new molecular biology (Luria, 1984; Corbellini, 1997; De Chadarevian, 2002; Morange, 2020). Between 1957 and 1962, Ageno worked on phage alpha in collaboration with the research group in microbiology of Sapienza University of Rome headed by Franco Graziosi. In the period between 1962 and 1969, with C. Frontali and E. Dore, Ageno set up an experimental research group in molecular biophysics with the aim of studying nucleic acids of phages and bacteria (see M. Ageno ‘I laboratori di fisica dell’Istituto Superiore di Sanità al 21 Marzo 1962’, in AA; see also Cassata, 2013, p. 97).

In the early 1960s, a number of researchers focused again on the question of what life is. As pointed out by Delbrück, the development of molecular biology made scientists ask the question slightly differently (Delbrück, 1976). In 1962, Delbrück asked Bohr to return to his ideas of 1932. Bohr rephrased his view and claimed that biology did not have to account for any atom of a living organism, but that biologists should proceed from the functional role of the parts of an organism to their physico-chemical structure (see Bohr, 1963a, b; Delbrück, 1976). Some years later, Delbrück recalled that Bohr’s view in 1932 entailed a sort of Heisenberg’s uncertainty principle for biology. As Einstein and Schrödinger hoped that sooner or later a return to the principles of classical mechanics would occur, so biologists and biochemists were not sympathetic with Bohr’s perspective on life (Delbrück, 1976). Taking life as a postulate was frustrating for a science whose goal was to explain what life is. Delbrück pointed out that the double helix discovery was for biologists what Einstein and Schrödinger were expecting for classical mechanics. He recalled the importance of Bohr’s lecture and its influence on his choice to focus on biology, but he also claimed that he was not going to address the epistemological question because: “this question is by now a dead issue in the area of ordinary biochemistry and physiology, and it has not yet become a live issue in the area of psychobiology” (Delbrück, 1976, p. 301; see also Kay, 1992). Delbrück pointed out that molecular biology now allows the study of organisms in terms of complex molecular systems. Returning to Bohr’s connection between life and light, he pointed to six molecules (chlorophyll, protochlorophyll, and retinal, cryptochrome, phytochrome and photoreactivating enzyme) whose photochemistry was related to life. He was still accepting Bohr’s view that life was to be taken as a postulate, but he suggested that the study of the photochemistry of the six molecules could shed some light on the origin of life (Delbrück, 1976).

Ageno disliked Bohr’s perspective, which he considered a form of vitalism because Bohr considered machines and living organisms as two very different kinds of objects (Ageno, 1986, pp. 297–298). Furthermore, he added that:

It is not, however, this basic vitalism that is the cause of the major distortions in Bohr’s biological thought, but rather the insistence with which, after the formulation of the *concept of complementarity*, he strove throughout the rest of his life to give it meaning and validity of cosmic scope (Ageno, 1986, p. 298).

In 1961, Wigner advanced his famous argument of ‘Wigner’s friend’, according to which the quantum description of objects is influenced by impressions entering consciousness, therefore quantum mechanics is incomplete until it includes consciousness (Wigner, 1961; on Wigner’s approach to quantum mechanics see Freire, 2007, 2009, 2010 and, 2015; on the Wigner paradox see Chalmers & McQueen, 2022). In 1965, Wigner and Landsberg stated that, as quantum mechanics cannot explain life, the notion of consciousness should be included therein. Ageno replied that, on the contrary, quantum mechanics did not contradict the possibility of life. Ageno thought that a description of the structure, nature and arrangement of the parts of an organism rather than its functions can shed light on life. Living organisms are made of atoms, so it is difficult to hold that quantum mechanics cannot explain them. Ageno argued that, for each of the peculiar functions of an organism (including reproduction), an equivalent model described by quantum mechanics can be found in the inorganic world. For instance, the growth of a bacterium in a suitable nutrient is analogous to that of an atomic nucleus in an environment with a thermal neutron density that is always different from zero. Wigner and Landsberg replied that Ageno disregarded the fact that, although self-reproductive processes were not limited to organic matter, the living state was a very uncommon state of matter. Wigner proposed writing a joint article with Ageno, who sent him a long paper with his reflections, but the project did not materialize (Ageno, 1965; Wigner & Landsberg, 1965; Wigner, 1969; see also Ageno, 1986, pp. 280–291). This is likely due to the loss of interest of the community of physicists in Wigner’s proposal. Although Wigner was convinced that his perspective was the genuine interpretation of Heisenberg and physicists started to talk of a Copenhagen interpretation and a Princeton interpretation, his view on consciousness was soon discarded and he was seen rather as a heterodox than as an orthodox physicist. Wigner was, nonetheless, among the first to deal with the quantum measurement problem (Freire, 2015).

## Biophysics as the physics of living systems

Over the years, Ageno refined his view of the relations between biology and physics. According to him, biophysics should be regarded as the physics of living systems, and the biophysicist is a physicist who knows biology (Ageno, 1987, pp. 79–99). Ageno not only immersed himself in the most significant biological theories but also encouraged his co-workers and students to explore a wide range of disciplines. A pupil and co-worker of his, Clara Frontali, recalled that he invited experts to deliver seminars on topics spanning from high energy physics to meteorology and oceanography (Frontali, 1993). Ageno distinguished between two different kinds of physical systems: bound systems and coherent systems. In a coherent system any piece is subordinated to a general functional design, whereas in a bound system it is the opposite. A crystal is a bound system, machines and living organisms are coherent systems. The simplest model of a living organism, a prokaryotic bacterial cell, is a coherent system having a programme. More complex forms of living organisms are just more articulated coherent systems with a programme; therefore, the researcher can study a simple model of a prokaryotic cell, and then extend it to more complex organisms (Ageno, 1987, pp. 86–90).

In July 1969, Ageno resigned from the ISS and in November 1970 he was appointed professor of biophysics at Sapienza University of Rome (‘Elenco delle pubblicazioni’, in AA, box 1, folder 1).^4^ Until 1978 Ageno’s research focused on the structure of the nucleic acid of the new bacteriophages isolated in polluted waters. This research was inconclusive, and its results were never published. According to Ageno, the reason for this was the impossibility of securing the required equipment. The university laboratory of La Sapienza had, indeed, fewer resources than the ISS, and Ageno had not succeeded in persuading the CNR (Consiglio Nazionale delle Ricerche), the National Research Council, to create a new institute devoted to the study of biological physics in previous years (M. Ageno ‘Promemoria sulle esigenze e il finanziamento di un istituto nazionale di fisica biologica’; M. Ageno ‘Promemoria sui compiti e sulla struttura di un istituto nazionale di fisica biologica (e sanitaria)’; ‘Relazione al Comitato per la Fisica del CNR’, in AA). Ageno had always dreamt of a laboratory devoted to the study of the physical principles underlying biology along the lines of the research programme he had found implicit in Schrödinger’s book. In 1964, when Adriano Buzzati-Traverso resigned as director of the LIGB (Laboratorio Internazionale di Genetica e Biofisica), International Laboratory of Genetics and Biophysics, Ageno was asked to take over, and he saw it as an opportunity to develop his perspective. Writing to Polvani, the president of the CNR, on which the LIGB depended, he proposed to transform it into an institute devoted to the study of the physical basis of biology. Polvani did not endorse Ageno’s project (see letter from M. Ageno to G. Polvani of 21 June 1964, in Archive of the CNR, Presidenza Polvani, box 22, folder 148; see also Cassata, 2013, pp. 306–308; on the LIGB see Capocci & Corbellini, 2002 and Cassata, 2013). Polvani’s decision is not surprising, because Ageno’s perspective of the relations between biology and physics was very different from that of molecular biology, whose scientific successes were undeniable, as Ageno himself acknowledged. Interestingly enough, biologists accused physicists of reductionism because they sought to reduce biology to physics. As highlighted by Michel Morange, biologists often made fun of physicists for trying to explain all living organisms, from bacteria and viruses to elephants, using the same principles, without understanding the complexity of the functioning of life (Morange, 2020, p. 53). On his part, Ageno considered molecular biology as a form of reductionism, because it reduced the complexity of living organisms to “a game of molecules” (Ageno, 1987).

As of 1979, Ageno focused on experimental research on the organization of the bacterial cell and developed a growing interest in the philosophical basis of Darwinism. The two projects were connected, as he tried to explain why bacteria tend to move away from each other in a liquid culture. For Ageno, this can only have an evolutionary explanation. He aimed to build a functional model of the simplest kind of organism as the first step towards his grand design of explaining the transition from non-living matter to a living organism by quantum mechanics (AA, ‘Elenco delle pubblicazioni’, box 1, series 1, folder 1; Ageno, 1987; Ageno, 1992a). For Ageno, Darwinian evolution was the key to understanding the difference between the living and the non-living world. In *La biofisica* he gave the following example. Let’s consider two systems: an *Escherichia coli* bacterium in a culture and a neutron of lanthanum in a nuclear reactor. Apparently, both evolve along the same path. The bacterium grows until it splits into two; the lanthanum atomic nucleus grows by absorbing slow neutrons inside the reactor, occasionally emitting an electron and a neutrino to maintain its internal equilibrium, until it splits into two nuclei of atomic mass of order of magnitude comparable to that of the original nucleus. There is, however, an important difference between the bacterium and the atomic nucleus of lanthanum: biological organisms may evolve by Darwinian natural selection, whereas non-living systems cannot. Thus, Darwin evolutionary theory, once reinterpreted in light of his physics of living organisms, became pivotal in Ageno’s view. Furthermore, according to Ageno, his perspective countered the accusation of circularity that had been levelled at Darwinism (Ageno, 1987, pp. 102–107; see also Ageno, 1986). Ageno also insisted that his perspective made it possible to transcend the incompatibilities between evolutionary and functional biology (Ageno, 1987; see also Cini, 1989 and, 1994). Molecular biology and evolutionary biology had been in conflict since the inception of the latter at both a theoretical and an institutional level: the growing power of the former threatened the latter. Molecular biologists did not seek the evolutionary meaning of biological processes, whereas evolutionary biologists always did (Smocovitis, 1992; Morange, 2020). For Ageno, his view of biophysics and the physics of living systems was the only way of unveiling the mystery of life. He was very critical of previous research by Alexander Oparin, Sidney Fox, and Graham Cairns-Smith on the pre-biotic broth, because their experiments were not derived from a precise conceptual framework; instead, “they are all models based on occasional ideas, suggested by crude superficial analogies, completely gratuitous proposals, lacking any foundation of general ideas, any theoretical conception to support them” (Ageno, 1987, p. 111; see also Ageno, 1986, pp. 390–393). Graham Cairns-Smith formulated the hypothesis that certain crystals may behave like primitive genes by transferring the disposition of their imperfections from one crystal to another (see Cairns-Smith, 1971 and, 1990). His perspective contrasted with Ageno’s view that crystals were bound systems, whereas living organisms were coherent systems.

According to Ageno, on the contrary, his biophysics was a hypothetical-deductive framework. It formulated some hypotheses from what we know about organisms, and then from these hypotheses it derived some experiments that could prove (or refute) the hypotheses. To understand the origin of life we should start from what we know about cells. Ageno stressed that his perspective was preferable because it made it possible to shed light on certain phenomena that biologists accept as primitive in the study of bacteria, such as that non-adaptive characters prevail and endure, or rapid speciation, which is incompatible with Darwinian gradualism (Ageno, 1986, pp. 393–394 and Ageno, 1987, p. 93).

For Ageno a living organism should be considered a “stream of energy in a valuable form that progressively degrades by organising materials from the environment to create structures useful for its own upkeep and maintenance” (Ageno, 1986, p. 182). According to him, his approach of looking at living organisms as coherent systems having a programme made sense of evolutionary theory. Ageno considered Ernst Mayr’s biological concept of species to be pivotal in the synthetic theory of evolution, although he was perfectly aware that in many cases other notions of species had to be used for the practical purpose of classifying species and that Mayr himself remarked that there were certain cases that did not fit with his theory (Ageno, 1986, pp. 22–25). But Ageno also remarked that for the aims of his biophysical approach, Mayr’s biological concept of species was not fully satisfactory because it did not induce classifications on the basis of the levels of complexity of organisms. For Ageno, classification of species on the basis of complexity might solve the problem of ring species or other difficulties such as the cases of populations that interbreed in certain geographical areas but not in others. Likewise, his approach might provide a better understanding of what a character is (Ageno, 1987, pp. 15–30).

In spite of what Ageno claimed, very few researchers looked at the whole matter in the same way he did. Ageno mentioned the approach of Mikhail Vladimirovich Volkenstein (Ageno, 1987, p. 38). Indeed, Volkenstein too regarded biophysics as “the science that studies life from the viewpoint of the laws and methods of experimental and theoretical physics” (Volkenstein, 1982, p. vii). Volkenstein exchanged letters with Bohr on the issue, but he agreed with Schrödinger in thinking that the understanding of life required new physical principles (Volkenstein, 1972, 1982). Like Ageno he considered that the Second Law of thermodynamics was only apparently contradictory with life and thought that “Wigner was mistaken because he did not take into account the ability of biopolymers (nucleic acids) to play the role of biosynthetic templates” (Volkenstein, 1982, p. 118).

In the late 1980s and early 1990s, Ageno focused on disseminating his epistemological views in a number of books in Italian. He likely thought that his ideas could be best appreciated by a younger generation of students who had not begun their scientific careers. In 1992, he published what he considered the most important contribution that he and his co-workers had made to biophysics, *La macchina batterica*, in which he proposed a quantum mechanics-based model for bacterial growth from a study of *Escherichia coli*. The book was published by a small firm after being rejected by major publishers such as Adelphi and Feltrinelli. As Giuseppe Tratteur stressed in his rejection letter, the book is three different books in one: a set of scientific papers that should first be published in an academic journal, a methodological book, and a didactic textbook (see letter from Feltrinelli’s editorial secretary of 20 September 1989 to M. Ageno and letter from G. Tratteur to M. Ageno of 21 July 1990, in AA).

In *Le origini dell’irreversibilità*, Ageno proposed an alternative solution to Boltzmann’s mechanical model of thermodynamics by considering the interaction of gas atoms with those of the recipient. His interests led him closer to Marcello Cini, a relativistic physicist, who since the early 1970s had been very critical of the scientific establishment and the current status of physics (see Ciccotti et al., 1976 and Cini, 1994; on Cini see Turchetti, 2016 and Ienna, 2020). In 1994, Cini published *Un paradiso perduto* (A Lost Paradise), which summarized his epistemological reflections. At that time Cini was focusing on epistemology, with a special interest in a non-reductionist view of the relations between biology and physics. There were many points in common between the two physicists of Sapienza University in Rome. Like Ageno, Cini engaged with the apparent tensions between the historicity of biology and the normativity of physics. He found in Darwinism a non-teleological description of scientific progress, and the key to non-reductionist explanations of a vast array of systems, including biological systems (Cini, 1994; see also Cini, 1989 and Somenzi, 1989). In *Un paradiso perduto*, Cini quoted Ageno’s view that Darwinism had become pivotal in biology, because molecular biologists, before many different possible equivalent explanations in terms of molecular structures, had to find the correct explanations in the evolutionary meaning of a given biological issue (Cini, 1994, pp. 117–118). Cini reviewed *Le origini dell’irreversibilità*. Ageno acknowledged his colleague and bitterly commented that he was the only one who took his work seriously. Cini was aware that Ageno’s reflections would not shake the scientific community. Indeed, in his review, Cini recalled that after Plank’s introduction of the notion of quanta, the puzzle had been largely solved. Rethinking an aspect that the scientific community no longer considered problematic did not attract the attention of the scientific community, which was much more interested in new theories and development (M. Cini, ‘Recensione a L’origine della reversibilità in l’Indice’; letter from M. Ageno to M. Cini, in AA).

## Conclusions

Mario Ageno was a complex figure in the panorama of twentieth-century scientific research. A number of molecular biologists came from physics and became interested in shedding light on the phenomenon of life because of their interest in quantum mechanics. Ageno was one of these physicists. He was one of the few researchers who tried to solve the mystery of life. Delbrück had operated within Bohr’s paradigm (and in dialogue with Bohr), while actively seeking a biological equivalent of the principle of complementarity. Gunther Stent, James Watson, and other eminent researchers had been intrigued by Schrödinger’s *What is Life?* Yet, Schrödinger’s ideas were for them a source of inspiration. By contrast, Ageno tried to develop Schrödinger’s ideas in an experimental programme. René Descartes thought that once the right method of investigation was found, it would be possible to easily find the solution to any scientific question that was legitimate to ask. Likewise, Ageno believed that the methodology of molecular biologists was wrong and that only a correct methodological approach would lead to an in-depth understanding of life. Translating a theoretical view into an experimental programme turned out, however, to be much more complicated than he expected, and Ageno did not achieve those major scientific breakthroughs that would attract a broader interest in his approach. Ageno authored a number of textbooks and popular books in which he aimed to disseminate his epistemological views. However, his very polemic writing likely did not help him to gain broader sympathy and consensus. Popular essays are, indeed, usually very captivating, whereas Ageno’s ones were quite polemic, especially with molecular biologists. His results, like those of all the scientists who engaged with the enormous task of solving the mysteries of life, did not satisfy him. Nonetheless, his perspective on the relations between physics and biology and his remarks on the relations between functional and evolutionary biology, including his idea of providing more rigorous definitions of the central concepts of Darwinism, remains an original and interesting epistemological position.

## Footnotes

1 H. J. Muller had discredited Delbrück’s model. But the importance of Schrödinger’s theses went beyond it (Fox Keller, 1990, p. 402).

2 The debate on the biological meaning of the Second Law of Thermodynamics dates back to its earlier formulation. In 1942, Joseph Needham had reviewed the literature stressing the significance of the principle for evolution. Needham stressed that a sharp distinction between the living and the non-living world was difficult to draw. At some levels, such as in the case of viruses, the two worlds intersect. Needham proposed a strong distinction between thermodynamic order and biological organization. See Needham, 1942.

3 Edoardo Amaldi reports that Ageno did not give up the walking stick he used in the laboratory until 1960 (E. Amaldi, ‘Gli Anni della Ricostruzione’, in Archives of the Department of Physics of Sapienza University of Rome, Archive of Amaldi Heirs, box 89, folder 3).

4 Ageno’s decision was a consequence of the upheaval of 1968, which had shaken the whole Italian academic system. Ageno had been criticized by a number of technicians and researchers for his rude manners and for the strict discipline he had imposed on them. He was disappointed when most of the workers in his laboratory failed to defend him (draft of a letter to the Minister of Health, in AA, folder 23, box 38).
